# Selective outcome reporting in paediatric dentistry restorative treatment randomised clinical trials—A meta‐research

**DOI:** 10.1111/ipd.13024

**Published:** 2022-07-26

**Authors:** Rokaia Ahmed Elagami, Tamara Kerber Tedesco, Claudio Mendes Pannuti, Gabriela Seabra da Silva, Mariana Minatel Braga, Fausto Medeiros Mendes, Daniela Prócida Raggio

**Affiliations:** ^1^ Department of Paediatric Dentistry, School of Dentistry University of São Paulo São Paulo Brazil; ^2^ Cruzeiro do Sul University São Paulo Brazil; ^3^ Department of Periodontics, School of Dentistry University of São Paulo São Paulo Brazil; ^4^ School of Dentistry Cardiff University Cardiff UK

**Keywords:** Paediatric dentistry, randomised controlled trials, restorative treatment, selective reporting

## Abstract

**Background:**

Selective outcome reporting (SOR) is a bias that occurs when the primary outcome of a randomised clinical trial (RCT) is omitted or changed.

**Aim:**

To evaluate the prevalence of SOR in RCTs on restorative treatment in primary teeth.

**Design:**

We conducted an electronic search on ClinicalTrials.gov and the World Health Organization platform (International Clinical Trials Registry Platform) on 1^st^ of April 2021, with no registry time or language restrictions. We included RCT protocols that evaluated restorative treatments in primary teeth and excluded trials that did not have a complete publication in a scientific journal. The chi‐squared test was used to identify the association between SOR and variables as a discrepancy in the follow‐up period, the timing of registration, the type of sponsorship and the type of study design (*α* = 5%).

**Results:**

Of the 294 identified protocols, 30 were included in the study. 83.3% of trials were registered retrospectively. SOR was observed in 53.3% (*n* = 16) of the published trials and was significantly associated with a discrepancy in the follow‐up period (*p* = .017).

**Conclusions:**

The high prevalence of SOR in RCTs on restorative treatment proves that this is a prominent threat. A proper preregistered protocol, declaration of any protocol deviation and allowance of stakeholders to compare the protocol with that of the submitted papers will achieve transparency.

**Why this paper is important to paediatric dentists**

- Restorative treatment trials in primary teeth that selectively modify outcomes of interest may distort the treatment effect.
- Practitioners should avoid performing restorative treatments in clinical practice based on misleading results.

## 1 INTRODUCTION

Dental caries in the primary dentition is considered the 10^th^ most common oral condition, affecting 621 million children worldwide.^1^ Moreover, untreated carious lesions in children negatively affect the oral health‐related quality of life^2^ and lead to growth and developmental problems.^3^ Well‐designed and well‐conducted randomised clinical trials (RCTs) are considered the best level of evidence for interventional studies.^4^, ^5^ The validity of clinical trials could be affected if they present biases,^6^ and selective outcome reporting (SOR) is considered a potential bias that can overestimate a study's effect.^7^

‘Spin’ or misrepresentation in scientific literature occurs when the authors distort the interpretation of results and mislead the readers by highlighting a specific treatment benefit despite a non‐significant difference in the primary outcome. Moreover, studies have shown that statistically significant results are more likely to be published than non‐significant (negative) results, leading to publication bias.^8^, ^9^ Consequently, some researchers tend to highlight the significant results, regardless of their non‐significant planned primary outcome, misleading the readers and jeopardising the clinical decision‐making.^10^

Since 2005, the International Committee of Medical Journal Editors (ICMJE) has declared that all clinical trials must be registered before enrolling the first participant. Consequently, the prospective registration of RCT protocols will help diminish reporting bias.^11^ Transparency between planned and published outcomes prevents wasting time, effort and money.^12^ If trials are well designed, conducted and reported faithfully, the results will contribute to scientific and clinical knowledge and be consistent with the ethical principle of equipoise. Two essential guidelines have been introduced to enhance the transparency of the protocols (‘Standard Protocol Items: Recommendations for Interventional Trials’) and published RCTs (‘Consolidated Standards of Reporting Trials)’.^13^, ^14^

To the best of our knowledge, there has been no previous evaluation of SOR in RCTs in paediatric dentistry. Thus, our primary aim was to examine SOR by comparing information from records with that from publications of RCTs concerning restorative dentistry in primary teeth. The secondary goals were to evaluate: (a) the introduction of new secondary outcomes (i.e., an outcome that was not described in the registry and that was introduced as a secondary outcome in the publication); (b) discrepancies between the trial registry and the publication regarding the study start date, number of arms, follow‐up period and sample size; (c) other types of discrepancies; and (d) the association between SOR and the timing of registration, the type of sponsorship, the type of study design and discrepancies in the follow‐up period.

## 2 MATERIALS AND METHODS

This study was a meta‐research study registered on the Open Science Framework platform (10.17605/OSF.IO/8H5PA). The research question for this study was: ‘From previously registered RCTs in restorative care for primary teeth, how many have a SOR in the published paper?’

### 2.1 Search strategy

We searched ClinicalTrials.gov and the World Health Organization (WHO) platforms (International Clinical Trials Registry Platform) on 1^st^ of April 2021, with no registry time or language restrictions, for RCT protocol registries that dealt with restorative treatment in primary teeth. The search strategy was as follows: (“dental caries” OR decay OR caries OR carious) AND (“primary teeth” OR “primary tooth” OR deciduous OR “primary dentition” OR children OR child OR infant). We limited the results of the ClinicalTrials.gov website to only ‘unknown and completed status’ protocols. We used two keywords for the WHO International Clinical Trials Registry Platform (ICTRP): “dental caries” and “primary teeth.” We checked the results of both searches to identify and eliminate duplicates.

### 2.2 Study selection

Two researchers (RAE and TKT) independently screened the titles and abstracts of registered protocols for eligibility. The inclusion criteria were as follows: (a) RCTs with two or more arms and (b) studies evaluating any restorative treatment in primary teeth. We did not include observational studies (cohort, case–control or cross‐sectional studies) or case series. We excluded any protocols that did not result in at least one publication in a peer‐reviewed, indexed, scientific journal. We searched for corresponding published articles for each included protocol by finding the reference(s) of the publication in the registry. When they were unavailable, we searched PubMed/MEDLINE and EMBASE using the principal investigator's name and protocol keywords. If we could not find any publication, we searched Google Scholar for the record number. If we found more than one publication, we extracted the data from the one with the same primary outcome. If we found more than one primary outcome publication, we extracted the data from the registry with the most extended follow‐up period. When no publication was found, we contacted the principal investigator and excluded the study protocol in case of no response. The reviewers were trained and calibrated by conducting a pilot screening of 10% of the retrieved articles. A third researcher (DPR) resolved any disagreement.

### 2.3 Data extraction

Two researchers (RAE and TKT) independently extracted data for each included protocol and its corresponding publication. Disagreements were solved by a third researcher (DPR). We registered all extracted data from the included protocols and publications in a standardised form.

We extracted information from the protocols, such as registry number, name and country of the principal investigator, primary registry date (and last time updated), study start date and completion date. Registry timing was analysed to determine whether the study was prospectively or retrospectively registered. If the protocol was registered before enrolling the first participant, it was considered a prospective registration, and we considered a retrospective registry if the authors recorded the protocol after enrolling the first participant. In addition, we identified whether there were any core changes in the protocol using the history of changes in ClinicalTrials.gov. We also extracted study funding (institutional or commercial) data, the number of arms and interventions, RCT design (parallel, split‐mouth, factorial or sequential), sample size, and follow‐up time. We also collected the number, nature and time frame for each primary and secondary outcome.

We gathered the number of publications, journal name, impact factor (Journal Citation Reports, 2020), study start date mentioned in the publication and paper publication date. We also recorded the funding for the article (institutional, commercial or incompletely declared). We classified the impact factor of the journals as ‘high impact’ or ‘low impact’, using the median as the cut‐off value. We recorded whether the authors declared the registry number in the published paper and whether they declared any deviation from the protocol. We also collected data on the number of arms and interventions, sample size and a priori sample size estimation based on the primary outcome. Regarding outcomes, we recorded the number, nature, time frame, and whether the primary or secondary outcomes showed statistical significance.

After collecting all data from protocols and publications, we extracted the discrepancies. We identified SOR according to a modification of Chan et al. (2004): (a) primary outcome in the registry reported as secondary in the publication (primary outcome downgrade); (b) secondary outcome in the registry reported as primary in the publication (secondary outcome upgrade); (c) a new primary outcome (i.e., an outcome that was not described in the registry) introduced in the publication; (d) primary outcome in the registry omitted in the publication; and (e) discrepancy in the primary outcome time frame (i.e., the timing of assessment of the primary outcome differed between the registry and the publication). When the registry had more than one primary outcome, we considered the primary outcome in the publication according to which the sample size was calculated, and all other outcomes were considered secondary outcomes. In addition, we investigated other discrepancies between the protocol and published articles, such as discrepancies in the study start date, study design, arms, sample size and sponsorship.

### 2.4 Data analysis

We used the Jamovi version 1.2 software to perform the statistical analysis. Quantitative variables are expressed as means and standard deviations, and qualitative variables are expressed as frequencies and percentages. We used the chi‐squared test to identify the association between SOR and the following variables:

- a discrepancy in the follow‐up period (yes or no),
- the timing of registration (retrospective or prospective),
- the type of sponsorship (incompletely declared, commercial or institutional) and
- the type of study design (parallel, split‐mouth, factorial or sequential).

The significance level for all analyses was set at 5%.

## 3 RESULTS

**FIGURE 1:**
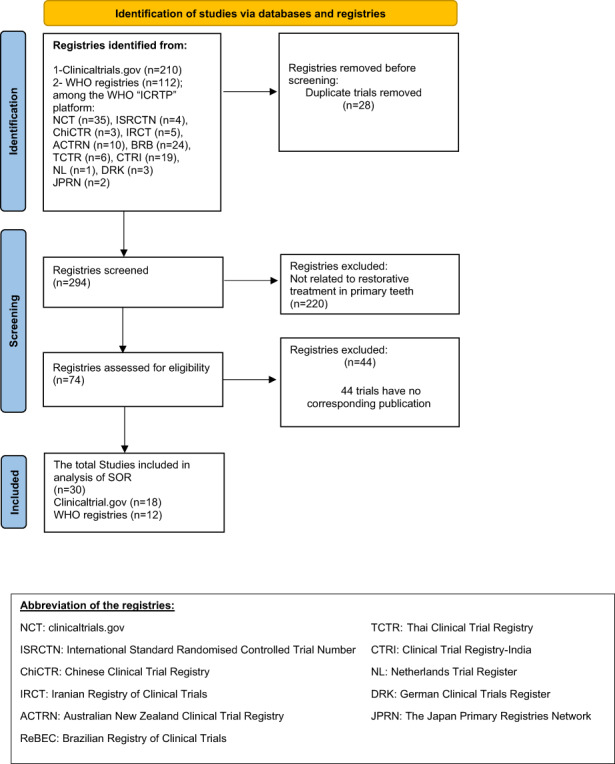
Flow chart of study selection. It represents the number of identified registries from both platforms (clinicaltrials.gov and WHO registries) with a statement for all abbreviations. It reports the number of trials after screening according to the eligibility criteria, and the total number of trials analysed.

Our search strategy identified a total of 322 protocols. After eliminating duplicates, 294 protocols were retrieved. After screening titles and abstracts, 74 protocols were included. Forty‐four protocols were excluded because we could not find their corresponding publications. We found 34 publications for the 30 trials, two focused on secondary outcomes, and two declared different follow‐up periods for the same trial. Following our aim to include only the primary outcome and the most extended follow‐up, 30 studies were eligible and included in the analysis. Figure 1 shows the study flowchart. We examined the agreement between reviewers using Cohen's kappa calculation, which resulted in a value of 0.84 (almost perfect agreement).

Table 1 presents the characteristics of the included protocols. Most protocols were funded by the principal investigator's institution and were registered retrospectively. Regarding the core changes in the protocol, most protocols had no listed modifications. Furthermore, most trials had two arms with parallel‐group designs. The primary outcome time frame ranged from 12 to 48 months, and more than half of the trials reported one primary outcome.

**TABLE 1:** Frequency distribution of all trial characteristics included in this study

Characteristics of publications	All trials (*n* = 30)
Location of the principal investigator, *n* (%)	
Europe	3 (10)
North America	1 (3.3)
South America	16 (53.3)
Asia	8 (26.7)
Africa	2 (06.7)
Study funding, *n* (%)	
Institutional funding	29 (96.7)
Commercial funding	1 (3.3)
Protocol core changes, *n* (%)	
No	1 (3.3)
Yes	9 (30)
No changes	13 (43.3)
Unclear^a^	7 (23.3)
Timing of registration, *n* (%)	
Prospective	5 (16.7)
Retrospective	25 (83.3)
RCT design^b^, *n* (%)	
Parallel	25 (83.3)
Split‐mouth	3 (10)
Factorial	1 (3.3)
Sequential assignment	1 (3.3)
Number of arms, *n* (%)	
≤2	20 (66.7)
≥3	10 (33.3)
Number of primary outcomes, *n* (%)	
1	24 (80)
≥2	06 (20)
Sample size	
Min–max	20–700
Mean (SD)	134.1 (154.7)
Period of follow‐up (months)	
Min–max	1–48
Mean (SD)	21.3 (10.6)
Time frame of the primary outcome (months)	
Min–max	12–48
Mean (SD)	23.5 (9.9)

Abbreviations: RCT, radomised clinical trial; SD, standard deviation; Min–max, the minimum value and maximum value.

^a^ The unclear results of protocol core changes due to some registry trials have no “history of changes” function.

^b^ The definitions of study designs are as follows: parallel—a type of study where two groups of treatments, A and B, are given so that one group receives only A, whereas another group receives only B; split‐mouth—a type of study where two groups of treatments, A and B, are given so that each side of the mouth (or quadrant) receives only A, whereas the other receives only B; factorial—a type of study whose design consists of two or more factors (treatments), each with discrete possible levels and whose subjects take all possible combinations of these levels across all such factors; and sequential assignment—a type of study that randomises participants into different sequences of intervention options based on a set of decision rules about when to adjust a participant's treatment.

Regarding the characteristics of the corresponding publications (Table 2), the trials were published in 19 different journals, mainly in paediatric dentistry. Most protocols were identified in one publication. Twenty‐seven studies (90%) cited registry numbers in their publications. Twelve (40%) papers showed statistically significant findings for a minimum of one primary outcome. Moreover, only two publications reported deviation from the registered protocol. Regarding the sample size, most authors estimated it based on the primary outcome. The primary outcome time frame ranged from 6 to 48 months.

**TABLE 2:** Characteristics of the corresponding publications included in this review

Characteristics of publications	All trials (*n* = 30)
Number of publications related to the protocol	
01	26 (86.7)
02 or more	4^a^ (13.3)
Registration number cited in the paper, *n* (%)	
No	2 (6.7)
Yes	27 (90)
Yes, without number	1 (3.3)
Change in protocol reported in the paper, *n* (%)	
No	28 (93.3)
Yes	2 (6.7)
Sample size calculation reported in the paper, *n* (%)	
No	5 (16.7)
Yes	25 (83.3)
Sample size calculation based on protocol primary outcome, *n* (%)	
No	13 (43.3)
Yes	17 (56.7)
Study funding, *n* (%)	
Incompletely declared	10 (33.3)
Institutional funding	17 (56.7)
Commercial funding	3 (10)
Number of primary outcomes, *n* (%)	
1	26 (86.7)
≥2	4 (13.3)
Sample size	
Min–max	24–728
Mean (SD)	139.9 (167.2)
Period of follow‐up (months)	
Min–max	6–48
Mean (SD)	19.5 (10.3)
Time frame of the primary outcome (months)	
Min–max	6–48
Mean (SD)	20.8 (10.8)
Study significance^b^, *n* (%)	
No	14 (46.7)
Yes	12 (40)
Unclear	4 (13.3)

*Note*: All the variables are represented in number (n) and percentage (%).

Abbreviations: SD, standard deviation; Min–max, the minimum value and maximum value.

^a^ Of the 4 trials that have more than one publication, two of them were with different follow‐up periods and the other two publications were for protocol's secondary outcomes.

^b^ The study showed significant results when a study reported statistically significant results for at least one of the primary outcomes. The study showed unclear results when the authors had not clearly described the statistical significance for the primary outcome.

SOR was observed in 16 (53.3%) trials (Table 3). Among the discrepancies between protocols and publications, the most common reason for SOR was a discrepancy in the primary outcome time frame (*n* = 12, 40%). Other discrepancies identified were as follows: primary outcome downgrade (*n* = 6, 20%); secondary outcome upgrade (*n* = 4, 13.3%); primary outcome reported in the protocol that was omitted from the publication (*n* = 1, 3.3%); and new primary outcome introduced in the publication (*n* = 3, 10%). Seventeen (56.7%) trials reported new secondary outcomes and various outcome discrepancies.

**TABLE 3:** Outcome discrepancies and distribution of general discrepancies identified by comparing the protocol and the corresponding publication

Characteristics	All trials (*n* = 30)
Trials with selective outcome reporting^a^	
No	14 (46.7)
Yes	16 (53.3)
Primary outcome downgraded, *n* (%)	
No	24 (80)
Yes	6 (20)
Secondary outcome upgraded, *n* (%)	
No	26 (86.7)
Yes	4 (13.3)
Primary outcome in protocol omitted in the publication, *n* (%)	
No	29 (96.7)
Yes	1 (3.3)
New primary outcome in the publication, *n* (%)	
No	27 (90)
Yes	3 (10)
Discrepancy in primary outcome time frame, *n* (%)	
No	18 (60)
Yes	12 (40)
New secondary outcome, *n* (%)	
No	28 (93.3)
Yes	2 (6.7)
Any other outcome discrepancy, including new secondary outcome	
No	13 (43.3)
Yes	17 (56.7)
General discrepancies^b^	
Discrepancy in start date, *n* (%)	
No	4 (13.3)
Yes	13 (43.3)
Incompletely declared	13 (43.3)
Discrepancy in number of arms, *n* (%)	
No	26 (86.7)
Yes	3 (10)
Incompletely declared	1 (3.3)
Discrepancy in sample size, *n* (%)	
No	15 (50)
Yes^c^	14 (46.7)
Incompletely declared	1 (3.3)
Discrepancy in the follow‐up period, *n* (%)	
No	19 (63.3)
Yes^d^	11 (36.7)
Discrepancy in sponsorship, *n* (%)	
No discrepancy	6 (20)
Institutional	13 (43.3)
Commercial	2 (6.7)
Incompletely declared	9 (30)
Discrepancy in study design, *n* (%)	
No	14 (46.7)
Yes	1 (3.3)
Incompletely declared	15 (50)
Total	30 (100)

^a^ According to the Chan et al. (2004) classification.

^b^ The general discrepancies are represented in number (n) and percentage (%). These discrepancies were collected by comparing the data from protocols with the data from publications.

^c^ Of the 14 studies that reported with a discrepancy in sample size, seven of them showed an increase in sample size and seven showed decreases in the sample size when comparing the protocol to the publication.

^d^ The discrepancy in the follow‐up period was identified when the author ends the study before or after the planned time frame. Of the 11 studies that reported with a discrepancy in the follow‐up, 9 of them reported also with SOR. Of these nine studies, six studies reported non‐significant results and three of them were completed earlier as the authors found a significant favored outcome.

Table 3 displays further discrepancies between protocols and publications. Thirteen studies (43.3%) reported a discrepancy in the study start date. Only three (10%) trials reported a discrepancy in the number of arms. Eleven trials had discrepancies in the follow‐up period, with seven decreasing and four increasing discrepancies. Thirteen trials reported the institutional difference in sponsorship, and two presented a commercial distinction between the registry and the publication.

Table 4 shows the association between SOR and discrepancies in the clinical trials. Only SOR was associated (*p* = .017) with a discrepancy in the follow‐up period. We calculated the median of the journals' impact factors (2.757) according to Journal Citation Reports (2020) and related the SOR to high‐impact or low‐impact factor journals. The impact factors of the journals ranged from 1.065 to 4.379. We found that SOR existed in four (25%) of the three journals with high‐impact factors (Clinical Oral Investigations, Journal of Dentistry and Caries Research). In addition, SOR was found in 12 (75%) of the 10 journals with low‐impact factors (Archives of Oral Biology, Quintessence International, Brazilian Oral Research, Pediatric Dentistry Journal, Laser in Dental Science, Journal of the Dental Association of Thailand, JDR Clinical & Translational Research, Journal of Clinical Pediatric Dentistry, Journal of Dentistry for Children and Alexandria Dental Journal). There was no statistically significant difference between SOR and the impact factor of the journals (*p* = .156).

**TABLE 4:** Association between selective outcome reporting and discrepancy in the follow‐up period, the timing of registration, the type of sponsorship in the publication and the type of study design in the protocol

Characteristics	SOR		Total	*p*‐value (chi‐squared test)*
	No	Yes		
Discrepancy in the follow‐up period, *n* (%)				
No	12 (40)	7 (23.3)	19 (63.3)	**.017** *
Yes	2 (6.7)	9 (30)	11 (36.7)	
Timing of registration^a^				
Retrospective	10 (33.3)	11 (36.7)	21 (70)	.873
Prospective	4 (13.3)	5 (16.7)	9 (30)	
Type of sponsorship, *n* (%)^b^				
Incompletely declared	3 (10)	7 (23.3)	10 (33.3)	.393
Institutional funding	9 (30)	8 (26.7)	17 (56.7)	
Commercial funding	2 (6.7)	1 (3.3)	3 (10)	
Type of study design, *n* (%)^c^				
Parallel	12 (40)	13 (43.3)	25 (83.3)	.177
Split‐mouth	0 (0)	3 (10)	3 (10)	
Factorial	1 (3.3)	0 (0)	1 (3.3)	
Sequential	1 (3.3)	0 (0)	1 (3.3)	

Abbreviation: SOR, selective outcome reporting.

* Chi‐squared test considering *p* < .05 significant level.

^a^ Retrospective: when the protocol was registered after the enrollment of the first participant. Prospective: when the authors recorded the protocol before the enrollment of the first participant.

^b^ Type of sponsorship as collected from corresponding publications.

^c^ Type of study design as declared at protocols.

## 4 DISCUSSION

This study is the first meta‐research to focus on SOR in paediatric dentistry restorative treatments and has shown a high prevalence of SOR. One systematic review in paediatric dentistry^15^ revealed that few good‐quality studies exist regarding the management of dental caries, and most have an increased risk of bias in providing evidence for strongly recommending the best treatment option. The suggested policy to overcome the SOR was trial registration with a pre‐specified list of all outcomes and transparent reporting of subsequent changes. Another attempt to improve outcome reporting is followed by some journals^16^ that have announced that each author must declare that the manuscript is an honest, accurate and transparent account of the study and that no critical aspect has been omitted.

The prevalence of SOR is still high and reported in the dentistry and medicine fields.^17^, ^18^ Similarly, our results proved the discrepancies between the registered protocols and published articles on restorative treatment in primary teeth. SOR was observed in 53.3% of the trials; more information regarding the reasons for SOR in each included trial is found in Table S1. Our results were similar to those of other studies, such as 48% in osteoarthritis trials,^19^ more than 55% in trials of dental implants^17^ and 53.8% in prospectively registered psychotherapy trials.^20^ A range of 40–62% has been reported in studies that evaluated publication bias and reporting bias in RCTs,^21^ where at least one outcome was omitted, introduced or changed. Empirical research pointed out that positive or statistically significant results are more likely to be published and negative results could be rejected.^22^ Thus, we suggest that the editors and reviewers accept negative results, considering that the authors followed the correct sample size estimation and proper methodological steps.

In our study, the primary outcome time frame discrepancy was most common among the trials reported with SOR. A recent study^19^ emphasised that 33% of their trials presented a primary outcome time point discrepancy. In haematological malignancies, a previous study^23^ assessed RCTs published in haematology journals and showed a change in the timing of assessment of the primary outcome by eight times (6.8%). In contrast to the study by Koufatzidou et al.^24^ which found an association between outcome reporting discrepancy and the type of study design, we could not find any association. A possible explanation for this finding is that most assessed trials did not clearly report the study design in their final publication.

Furthermore, we found a significant association between SOR and discrepancies in the follow‐up period. Accordingly, trials that differed in follow‐up periods from their corresponding publications presented outcome bias. Corroborating these results, Rosati et al.^25^ found that 13 RCTs were completed earlier than expected without justification. Our study observed 11 studies that had a discrepancy in the follow‐up period; two had no SOR. Seven studies changed the primary outcome time frame, and two misreported their primary outcomes (i.e., they probably occurred when the authors decided to end the study before or after the completion of follow‐up periods, either if they had non‐significant results [*n* = 6 studies] or if the favoured result was found to be significant [*n* = 3 studies]), affecting the study's reliability.

Although there was no association between SOR and the type of sponsorship, 43.3% of the trials had institutional discrepancies. Consequently, some of these trials have shown one or more reasons for SOR; one study reported similar results.^7^ Also, 14 studies presented a discrepancy in sample size, as observed in other studies.^24^, ^25^ Among these 14 trials, seven showed a decrease in the sample size, and seven trials had increased the sample size in the publication.

There was no association between SOR and the registry timing (retrospective or prospective). The percentage of retrospectively registered trials was, however, high (83.3%), which has also been observed in other publications.^18^, ^20^, ^26^ Furthermore, we verified that 14 trials with SOR were retrospectively published in 11 different journals (three were ICMJE member journals). Since 2005, all authors must follow the ICMJE policy, which advocates registering the protocol with pre‐specified and clear outcomes before starting the trials (before enrolling the first participant).^11^ More data on the journals' policies are depicted in **Table** **S2**.

We expanded our search to two different platforms to increase the number of included protocols. Consequently, we had a limitation that the protocols retrieved from ICTRP have no ‘history of changes’ function. Moreover, we noticed that inappropriate registration of the trials and unclear data on outcome measures could affect the recognition of outcome discrepancies, lowering the prevalence of SOR in our study. Another limitation of our study is that the protocol was excluded when we did not obtain a response from the corresponding or leading author. Although most of the publication references in the registries were not linked to the protocol registry, we overcame this problem by searching manually for the registration number on Google Scholar to find the corresponding publications. Therefore, authors should ensure declaring and linking the registration number in the publication to be automatically indexed in the registry to be quickly recognised by readers and peer reviewers.

Despite all attempts to decrease SOR and increase transparency, our findings indicate that the prevalence of SOR is high in trials focusing on restorative treatment in primary teeth, which are published in either high‐impact or low‐impact journals. Furthermore, editors, authors and reviewers can prevent SOR through joint attempts. Editors and reviewers should request the protocol registry to assess discrepancies in the time frame or outcomes and the journals' adherence to the reporting guidelines. Moreover, the authors should comply with the reporting guidelines and report any deviations from the protocol in their publication.^14^, ^27^, ^28^ Consequently, this type of bias could pose a significant threat to policymakers and clinicians. Moreover, sponsorship funding should be declared correctly, and editors should ensure that funded trials maintain their pre‐specified registered protocol.

Of the 74 potential registries included in the methodological review, we could only find 34 complete publications (two had 1‐year and 2‐year results published, and two others had published the primary outcome separate from the secondary outcomes). After contacting the authors, nine replied with the reasons for not publishing the data, and 35 authors did not respond. Among the authors' answers, six manuscripts were submitted for publication, one trial had no results, one was withdrawn, and one was under analysis. We assume that the 35 non‐responded trials were not published because they had problems with the design and conduct or even negative results. All information from an RCT must be available to reduce research waste and well‐designed RCTs conducted with negative results must be published in high‐quality journals to minimise publication bias.^29^ We hypothesise that the publication bias could lead the authors to report an outcome selectively (though not deliberately), as the research community tends to avoid publishing negative results.^30^

In this sense, we can conclude that SOR in paediatric dentistry restorative trials is high and might impact the clinician's decision‐making regarding primary tooth's restorative treatment. Therefore, there is still a need to enhance trial registration awareness and properly pre‐specify the outcomes. Consequently, we recommend further investigation of the publication bias in paediatric dentistry.

## AUTHOR CONTRIBUTIONS

D.P.R., C.M.P. and F.M.M. conceived the idea; R.A.E., T.K.T. and D.P.R. collected the data; R.A.E., T.K.T. and D.P.R. analysed the data; R.A.E. and G.S.S. drafted the manuscript; D.P.R., M.M.B., F.M.M. and C.M.P. revised and gave final approval of the manuscript.

## CONFLICT OF INTEREST

None of the authors have any financial interests related to the article.

## Supporting information


Table S1

Table S2

## Data Availability

The data that supports the findings of this study are available in the supplementary material of this article
