# Supplementary material for: Selective outcome reporting in paediatric dentistry restorative treatment randomised clinical trials—A meta‐research
Source: Int J Paediatr Dent. 2022 Jul 26;33(1):89–98. doi: 10.1111/ipd.13024 (PMC10087835; doi:10.1111/ipd.13024)
Supplement: Supplementary file 1 — Table S1 [file IPD-33-89-s001.docx]

Supplementary 1. This table represent a part of data sharing for all the retrieved trials with their corresponding publications to report the reasons for SOR, timing of registration, discrepancy in study start date, discrepancy in follow up period, discrepancy in sponsorship by comparing the data collected from the protocol to the data collected from publication.

| **Registration numbers, and web link for the 34 RCTs published (first author and publication**  **reference) ^[[1]](#footnote-1)^** | **Timing of registration** | **Discrepancy in study start date** | **Discrepancy in follow‐up period** | **Discrepancy in sample size** | **Discrepancy in the sponsorship** | **Primary outcome downgrade or omitted or secondary outcome upgraded** | **New 1ry outcome** | **Discrepancy in 1ry outcome time frame** | **SOR** |
| --- | --- | --- | --- | --- | --- | --- | --- | --- | --- |
| NCT04265833, URL  <https://clinicaltrials.gov>    (Sahin N et al. 25(6):3945-3955;Jun 2021) | (retrospective)  Registered in February 2020, the study start date in the registry was February 2015 | Incomplete declared in the RCT | - | - | - | - | - | - | - |
| NCT03756025, URL  <https://clinicaltrials.gov>  (Faustino-Silva, D.D., et al. **23,**3721–3729 2019) | (retrospective)  Registered in November 2018, the study start date in the registry was June 2008 | incomplete declared in the RCT | - | - | Institute discrepancy. In the registry was hospital Nossa Senhora da Conceição but in the RCT was Federal University of Rio Grande  do Sul | - | - | in the registry was 12 months, in the publication 48 months | Yes |
| NCT03220360, URL  <https://clinicaltrials.gov>  (Ma Xiao-hong et al.  21(22): 34943500  2017) | (retrospectiv)  Registered in July 2017, the study start date in the registry was June 2016 | In the registry was June 2016, but in the publication from September 2016 | - | - | Institute discrepancy  In the registry was Chengdu Maternal and Children's Health Care Hospital, but in the publication was the Popular Application Project of Health and Family Planning Commission of Sichuan Province. | - | - | - | - |
| NCT03100773, URL  <https://clinicaltrials.gov>  (Salas Huamani JR et al. 97:191-197  Oct 2018) | (retrospective) Registered in April2017, the study start date in the registry was January 2015. | incomplete declared in the RCT | - | - | Institute discrepancy, in the protocol did not declare CAPES | Since they have 6 primary outcomes in the registry, we consider the primary outcome according to sample calculation. In RCT, the sample calculation is based on salivary biomarkers and HR. They have upgraded the cognitive and behavioral analysis and downgrade the salivary biomarkers and HR | - | - | Yes |
| NCT03030690, URL  <https://clinicaltrials.gov>  (El-Housseiny AA et al. 50(7):522-532  2019) | (retrospective)Registered in January 2017 , the study start date in the registry was November 2016. | In the RCT October 2015 but in the registry November 2016 | In the registry 6,12,18, 24 months but in the RCT 6 and 12 months | - | - | - | - | In the registry 24 months but in the RCT 12 months | Yes |
| NCT02969538, URL  <https://clinicaltrials.gov>  (Cavalheiro CP et al. 34, e081  2020) | (prospective) Registration date was at the same month of the study start date in the registry (November 2016) | In the registry was November 2016 , but in the RCT was April 2016 | - | In the registry was 70 , but in the RCT was 130. | Institute discrepancy. The protocol did not declare the CNPq and CAPES. | - | - | - | - |
| NCT02799927, URL  <https://clinicaltrials.gov>  (Garrocho-Rangel A  39(5):377-382  Sep 2017) | (retrospective)Registered in June 2016, the study start date in the registry was November 2014. | incomplete declared in the RCT | In the registry 3,6,12,18 and 24 months , but in the RCT 1,3,6,12 months. | - | incomplete declared in the RCT | the primary outcome in the registry was pain (presence/ absence) it downgrades and replace by new primary outcome (success rate) in RCT that join all the primary and secondary outcomes from the registry | In the registry only pain (presence/  absence) is the primary outcome, but in the RCT was clinical and radiographic success rate. | In the registry 24 months, but in the RCT was 12 | Yes |
| NCT02734420, , URL  <https://clinicaltrials.gov>  (Costa-Santos, L. et al. **3,**275–281 November 2019) | (retrospective)Registered in April 2016, the study start date in the registry was September 2013. | In the registry was September 2013 but in the RCT was October 2016 | In the registry it was 6,12 and 24 months, but in the RCT it was 3,6 and 9 months. | - | Institute discrepancy, in the registry did not include FAPESP as in the RCT | - | - | In the registry 24 months, but in the RCT 48 and 72 hour | Yes |
| NCT02569047, URL  <https://clinicaltrials.gov>  (Araujo, M.P. et al **20,**318  2020) | (prospective) Registered in October 2015, the same month of study start date in the registry | - | - | The sample size was 131 in the registry, but 124 in the RCT | Institute discrepancy, in the registry did not include FAPESP, CNPq and CAPES | - | - | - | - |
| NCT03855527, URL  <https://clinicaltrials.gov>  (Matar, L. et al. adjalexu.2020.32224.1074) | (retrospective)  Registered in February 2019, the study start date in the registry was January 2018 | In the registry was January 2018, but in the study was February 2018 | - | In the registry was 60, but in the RCT was 40. | Institute discrepancy, in the registry was personal funding but in the RCT declared no specific funding | Since they have 6 primary outcomes in the registry, we consider the primary outcome according to sample calculation. In RCT, the sample calculation based on reduction in mean (SD) of log transformed  TVC after 2 weeks. They have upgraded the baseline microbiological assessment in each group and compared the two groups. Also, downgrade the reduction of the bacterial count after 14 days | - | - | Yes |
| NCT02437565, URL  <https://clinicaltrials.gov>  (Pani SC et al. 16(6):461-6  Dec 2015) | (retrospective) Registered In May 2015, the study start date in the registry was August 2013. | In the RCT was April 2013 but in the registry August 2013 | - | - | incomplete declared in the RCT | - | - | - | - |
| NCT02377297, URL  <https://clinicaltrials.gov>  (Olegário IC et al. 57:45-50  Feb 2017)  **(1 year publication)** | (retrospective) registered March 2015, the study start date in the registry November 2014 | In the RCT was October 2014 but in the registry was November 2014 | - | - | Commercial discrepancy as in the RCT (DFL company) wasn’t declare in the registry | - | - | In the registry 2 years but in the RCT was 1 year | yes |
| NCT02377297, URL  <https://clinicaltrials.gov>  (Olegário IC et al.  101:103446  October 2020)  **(2 years publication was included)** | (retrospective) registered March 2015, the study start date in the registry November 2014 | In the RCT was October 2014 but in the registry was November 2014 | In the RCT was 2,6,12 and 24 months but in the registry 12 months | - | Commercial discrepancy as in the RCT (DFL company) was not declared in the registry | - | - | - | - |
| NCT02232828, , URL  <https://clinicaltrials.gov>  (Elhennawy K et al. 77:72-77  Oct 2018)  **(1 year publication)** | (prospective) Registered September 2014, the study start date in the registry was October 2014 | Incomplete declared in the registry | In the registry was 36 months but in the RCT was 12 months | In the registry was 300 but in the RCT was 74 | Institute discrepancy | - | - | In the registry was 36 months but in the RCT was 12 months | Yes |
| NCT02232828, , URL  <https://clinicaltrials.gov>  (Elhennawy K et al.  25(2):645-652  February 2021)  **(2 years publication was included)** | (prospective) Registered September 2014, the study start date in the registry was October 2014 | Incomplete declared in the registry | As they declare in the RCT it was an interim publication with 24 months so, **No discrepancy** | In the registry was 300 but in the RCT was 74 | Institute discrepancy | - | - | As they declare in the RCT it was an interim publication with 24 months so, **No discrepancy** | - |
| NCT02217098, URL  <https://clinicaltrials.gov>  (Olegário, I.C et al. **23,**1761–1770  2019) | (retrospective) registered August 15, 2014. The study start date in the registry July 2014 | In the registry was July 2014, but in the RCT was October 2013 | In the registry was 1,6,12,18 and 24 months. but in the RCT was 2,6,12,18, 24 and 36 months | In the registry there were 530 participants, but in the RCT there were 568 participants. | Commercial discrepancy as they declared in RCT Dentsply and GC Europe for supplying the material that was not declared in the registry | - | - | - | - |
| NCT02093091, URL  <https://clinicaltrials.gov>  (Abo-Hamar SE et al. 46(5):381-8  May 2015) | (retrospective) registered March 2014, the study start date in the registry June 2011 | incomplete declare in the RCT | - | - | incomplete declared in RCT | - | - | - | - |
| NCT02903979, URL  <https://clinicaltrials.gov>  (Waller MV et al.  88(1):52-57  Jan 2021) | (prospective) registered September 2016 , the study start date in the registry was November 2016 | In the registry was November 2016 but in the RCT was April 2016 | In the registry  6,12,24 months. But in the RCT was before and after the treatment | In the registry was 108 but in the RCT was 26. | Institute discrepancy | The pulp vitality which was primary outcome in the registry omitted in the publication | New primary outcome introduced “mean  change in GBI values before and after dental treatment.” | In the registry 24 months but in the RCT 6 months. | Yes |
| NCT01797458 , URL  <https://clinicaltrials.gov>  (Santamaría RM et al.  51(6):605-614  Dec 2017) | (retrospective)  Registered in February 2013, the study start day in the registry was May 2011 | Incomplete declared in the RCT | It was24 months, but in the RCT 2.5 years | - | - | - | - | In the registry was 2 years but in the RCT 2.5 years | Yes |
| NCT01449136, URL  <https://clinicaltrials.gov>  (Ferreira JM et al.  24(1):68-73  2013) | (retrospective)  Registered in October 2011, the study start date in the registry was January 2008 | incomplete declared in the RCT | - | - | Incomplete declared in the RCT | - | - | - | - |
| ChiCTR2000032462, URL <http://www.chictr.org.cn/enindex.aspx>.  ( Xiaoxian Chen et al.  **25,**3067–3076  2021) | (retrospective) Registered in April 2020, the study start date in the registry was January 2017 | - | - | In the registry 118, but in the RCT was 175. | - | - | - | - | - |
| RBR-9kkv53 , URL <https://ensaiosclinicos.gov.br/>  (Hesse, D et al.  **14,**58  2014) | (retrospective) Registered December 2013, the study start date in the registry January 2010 | In the registry was January 2010, but in the RCT was from 2007 | - | - | Institute discrepancy as in the RCT declared CNPq and FAPESP but in the registry was faculty of dentistry, university of São Paulo | - | - | - | - |
| RBR-4nwmk4 , URL <https://ensaiosclinicos.gov.br/>  (Moura MS et al.  24;33:e125  Jan 2020) | (retrospective) Registered January 2016, the study start date in the registry was September 2015 | Incomplete declared in the RCT | - | In the registry was 700 but in the RCT was 728. | Incomplete declare in the RCT | The loss of restorations was upgraded which was a secondary outcome in the registry. The success of the restoration was downgrade which was primary outcome in the registry | - | - | Yes |
| RBR-5sb8sb , URL  <https://ensaiosclinicos.gov.br/>  (Stafuzza TC et al.  3;27:e20180700  Jun 2019) | (retrospective) Registered March 2016, the study start date in the registry was September 2015 | Incomplete declared in the RCT | - | In the registry was 93, but in the RCT was 36. | Institute discrepancy as in the registry university and CAPES, but in the RCT was FAPESP | - | - | - | - |
| NCT03657862, URL <https://clinicaltrials.gov>  (Jiang M et al.  100:103435  SEP 2020)  **(Primary outcome publication was included)** | (retrospective)  Registered September 2018, the study start date in the registry was September 2017 | In the registry was September 2017, but in the RCT was October 2017 | - | In the registry was 195, but in the RCT was 194 | - | The Primary outcome which was the success of the ART restoration was downgraded and a new primary outcome which is time used to place the restoration was introduced. | The time used to place restoration had been introduced to the results. **Note**: it was declared in the RCT as secondary but not in the registry | - | Yes |
| NCT03657862, URL <https://clinicaltrials.gov>  (Jiang M et al.  88:103171  September 2019  **(Secondary outcome publication)** | (retrospective)  Registered September 2018, the study start date in the registry was September 2017 | Incomplete declared in the RCT | In the registry was 24 months, in the RCT was baseline and 6 months | In the registry was 195, but in the RCT was 194 | - | - |  | In the registry the timeframe of secondary outcomes were 24 months but in the RCT was baseline and 6 months | Yes |
| RBR-954xsg, URL <https://ensaiosclinicos.gov.br/>  (Sarti CS et al.  15;42(2):110-115  Mar 2020) | (retrospective) Registered in April 2016, the study start date in the registry was May 2015 | In the registry was May 2015, but in the RCT started from 2014 | In the registry was 36 months, but in the RCT was 24 months | - | Incomplete declare in the RCT | - | - | In the registry was 36 months but in the RCT was 24 months | Yes |
| RBR-2t54nx, URL <https://ensaiosclinicos.gov.br/>  (Polizeli SAF  10(2):108-116  Feb 2019) | (retrospective)Registered in October 2018, the study start date in the registry was February 2013 | Incomplete declared in the RCT | - | In the registry was 20, but in the RCT was 24. | Institute discrepancy, in the registry CAPES and FAPESP, but in the RCT was CAPES, FAPESP and CNPq | - | - | - | - |
| TCTR20170609002, URL http://www.thaiclinicaltrials.org/#  (Krongkan Thongrakkhao et al.  69(2):184-196  Jan 2019) | (retrospective) Registered in June 2017, the study start date in the registry was August 2016 | Incomplete declare in the RCT | - | Incomplete declare in the registry | Incomplete declare in the registry | The radiographic examination was upgraded in the RCT which was secondary in the registry. The clinical evaluation was downgraded which was primary outcome in the registry | - | In the registry was 6 and 12 months but in the RCT was 6 months | Yes |
| NCT03063307, URL <https://clinicaltrials.gov>  (Vollú AL et al.  88:103165  Sep 2019)  **(Primary outcome publication was included)** | (retrospective) Registered in February 2017, the study start date was September 2016 | In the registry was September 2016, but in the RCT from June2016 | - | In the registry 119, but in the RCT 118 | Institute discrepancy  In the registry university but in the RCT was FAPERJ | - | - | In the registry was 48 months but in the RCT was 12 months | Yes |
| NCT03063307, URL <https://clinicaltrials.gov>  (Rodrigues GF et al.  42(5):373-379  September 2020)  **(Secondary outcome publication )** | retrospective) Registered in February 2017, the study start date was September 2016 | In the registry was September 2016, but in the RCT from June2016. They have declared the questionnaires before the treatment . | - | In the registry 119, but in the RCT 118 | Institute discrepancy  In the registry university but in the RCT was CAPES | - | - | - | - |
| RBR-4qdx3v , URL <https://ensaiosclinicos.gov.br/>  (de França Lopes CMC  et al.  15;40(2):98-104  Mar 2018) | retrospective) Registered in July 2018, the study start date in the registry was March 2016 | Incomplete declare in the RCT | In the registry was 24 months, but in the RCT was 12 months | In the registry 23 participants but in the RCT was 33 participants | Incomplete declared in the RCT | - | - | In the registry was 3,6,12,24 months but in the RCT was 6 and 12 months | Yes |
| ACTRN12614000844640, URL <https://www.anzctr.org.au/Default.aspx>  (Boyd DH et al.  6(2):205-212  Apr 2021) | (prospective)  Registered in August 2014, the study start date in the registry was July 2014. It was registered within 1 month | - | In the registry was 36 months, but in the RCT was 24 months | In the registry was 150, but in the RCT was 295 | - | - | - | In the registry was 36 months, but in the RCT was 24 months | Yes |
| CTRI/2013/06/003714, URL <http://ctri.nic.in/Clinicaltrials/login.php>  (Mittal HC et al. 40(5):345-52  2016) | (retrospective) Registered in June 2013 , the study start date in the registry was December 2010 | - | In the registry 6 and 12 months, but in the RCT every 6 till 36 months | - | Incomplete declare in the RCT | - | - | In the registry was 3,6 and 12 months but in the RCT 36 months | Yes |

1. **Note:** we have extracted the data from the 34 published article but included in the analysis only 30 “with the same primary outcome and longest follow up “. We have extracted the data for these 4 publications to decide which is the same primary outcome or/and longest follow up periods. [↑](#footnote-ref-1)
