# Supplementary material for: Selective outcome reporting in paediatric dentistry restorative treatment randomised clinical trials—A meta‐research
Source: Int J Paediatr Dent. 2022 Jul 26;33(1):89–98. doi: 10.1111/ipd.13024 (PMC10087835; doi:10.1111/ipd.13024)
Supplement: Supplementary file 2 — Table S2 [file IPD-33-89-s002.docx]

Supplementary 2. Transparency policies for the journals that have publications included in our study.

| Journal title | Transparency Policies |
| --- | --- |
| 1. Clinical Oral investigations^[[1]](#footnote-1)^ | 1. To ensure the integrity of the reporting of patient-centered trials, authors must register prospective clinical trials (phase II to IV trials) in suitable publicly available repositories. For example [www.clinicaltrials.gov](http://www.clinicaltrials.gov/) or any of the primary registries that participate in the [WHO International Clinical Trials Registry Platform](http://www.who.int/ictrp/network/primary/en/). 2. For clinical trials that have not been registered prospectively, authors are encouraged to register retrospectively to ensure the complete publication of all results. The trial registration number (TRN), date of registration and the words 'retrospectively registered’ should be included as the last line of the manuscript abstract. |
| 1. Archives of Oral Biology | - The Journal has declared that the clinical papers should follow these guidelines generally follow the (Uniform Requirements for Manuscripts Submitted to Biomedical Journals). Registration of clinical trials in a public trial registry at or before the time of first patient enrollment as a condition of consideration for publication. |
| 1. QUINTESSENCE INTERNATIONAL | - No policy was found for the clinical trials except that (Report clinical trials using the CONSORT guidelines) |
| 1. Pediatric Dentistry Journal^[[2]](#footnote-2)^ | - Manuscript submission guidelines for Pediatric Dentistry follow the ‘uniform requirements for manuscripts submitted to biomedical journals’ which have been developed by the International Committee of Medical Journal Editors (ICMJE). Please visit the ICMJE website at: “http://www.icmje.org/ manuscript_1prepare.html” for more information. - Acknowledgment: Funding and other sources of support must be disclosed in the acknowledgment section. |
| 1. Lasers in Dental Science^[[3]](#footnote-3)^ | 1. To ensure the integrity of the reporting of patient-centered trials, authors must register prospective clinical trials (phase II to IV trials) in suitable publicly available repositories. For example [www.clinicaltrials.gov](http://www.clinicaltrials.gov/) or any of the primary registries that participate in the [WHO International Clinical Trials Registry Platform](http://www.who.int/ictrp/network/primary/en/). 2. For clinical trials that have not been registered prospectively, authors are encouraged to register retrospectively to ensure the complete publication of all results. The trial registration number (TRN), date of registration and the words 'retrospectively registered’ should be included as the last line of the manuscript abstract. |
| 1. Journal of Dentistry^[[4]](#footnote-4)^** | - Registration in a public trials registry is a condition for publication of clinical trials in this journal in accordance with International Committee of Medical Journal Editors recommendations. Trials must register at or before the onset of patient enrolment. |
| 1. Caries Research^[[5]](#footnote-5)^†† | 1. The presentation of manuscripts should follow the Uniform Requirements for Manuscripts Submitted to Biomedical Journals from the International Committee of Medical Journal Editors (ICMJE). 2. Karger follows the [WHO definition](https://eur02.safelinks.protection.outlook.com/?url=https%3A%2F%2Fwww.who.int%2Fictrp%2Fen%2F&data=02%7C01%7Cauthor-guidelines%40karger.com%7Cc64189b1f9784c4e708608d7573a5fa5%7C69e7eb606e904a0590b15b8d6d697087%7C0%7C0%7C637073780550190367&sdata=%2B%2BC%2FMxqRNwR7yjc1%2Fakijjtk3A0YK8ybuGnIb4MprkU%3D&reserved=0) of clinical trials *"A clinical trial is any research study that prospectively assigns human participants or groups of humans to one or more health-related interventions to evaluate the effects on health outcomes [...] Interventions include but are not restricted to drugs, cells and other biological products, surgical procedures, radiologic procedures, devices, behavioural treatments, process-of-care changes, preventive care, etc. This definition includes Phase I to Phase IV trials.”* |
| 1. Brazilian Oral Research | - Clinical Trials according to the CONSORT guidelines, available at [www.consort-statement.org](http://www.consort-statement.org/). The clinical trial registration number and the research registration name will be published along with the article. |
| 1. Journal of The Dental Association of Thailand | - They have no policy against Selective Outcome Reporting. |
| 1. JDR Clinical & Translational Research Instructions for Manuscript Submission | - Manuscript submission guidelines for the JDR Clinical & Translational Research follow the “Uniform Requirements for Manuscripts Submitted to Biomedical Journals” set forth by the International Committee of Medical Journal Editors (ICMJE). For additional information please visit the ICMJE web site at http://www.icmje.org/. |
| 1. Journal of Clinical Pediatric Dentistry | - They have no policy against Selective Outcome Reporting. |
| 1. Journal of Dentistry for Children | - Manuscript submission guidelines for Pediatric Dentistry follow the ‘uniform requirements for manuscripts submitted to biomedical journals’ which have been developed by the International Committee of Medical Journal Editors (ICMJE). Please visit the ICMJE website at: “http://www.icmje.org/ manuscript_1prepare.html” for more information. |

1. **NOTE:** since they accept the retrospective registered trials, but the authors must include “retrospective registered” in the manuscript. For the included trial in our study (registry number NCT03756025) the authors did not declare that it was retrospectively published. [↑](#footnote-ref-1)
2. **NOTE:** all the included trials in our study have not reported the source of funding in their articles, registries number (NCT02799927, RBR-954xsg, and RBR-4qdx3v). [↑](#footnote-ref-2)
3. **NOTE:** since they accept the retrospective registered trials, but the authors must include “retrospective registered” in the manuscript. For the included trial (registry number NCT02734420) in our study the authors did not declare that it was retrospectively published. [↑](#footnote-ref-3)
4. ** **NOTE:** Although they have declared that it is mandatory for prospectively registration, they accepted retrospective trials which included to our study ( registry number NCT03657862 and NCT03063307) and there was Selective Outcome Reporting. [↑](#footnote-ref-4)
5. †† **NOTE: T**he included trial (NCT01797458) had been published in Caries Research and it was retrospectively registered. Also, the trial reported a selective outcome because of discrepancy in primary outcome time frame. [↑](#footnote-ref-5)
